# Overlapping promoter library designed for rational heterogenous expression in *Cordyceps militaris*

**DOI:** 10.1186/s12934-022-01826-0

**Published:** 2022-06-02

**Authors:** Mengdi Lyu, Jiapeng Zeng, Yue Zhou, Tongyu Zhang, Aiping Wang, Jiezhao Ma, Ziyi Wu, Alvaro Castells-Garcia, Esther González-Almela, Junfang Lin, Tao Wei

**Affiliations:** 1 Department of Bioengineering, College of Food Science, South China Agricultural University, Guangzhou, 510640 Guangdong China; 2 Research Center for Micro-Ecological Agent Engineering and Technology of Guangdong Province, Guangzhou, 510640 China; 3 Bioland Laboratory, Guangzhou, 510005 China

**Keywords:** *Cordyceps militaris*, Fungi, Overlapping promoter, BioBricks

## Abstract

**Background:**

*Cordyceps militaris*, a kind of edible and medicinal fungus widely accepted in East Asia, has attracted much attention as a potential cell factory for producing adenosine analogs. Despite the rapid development in gene editing techniques and genome modeling, the diversity of DNA elements in *C. militaris* was too short to achieve rational heterogeneous expression for metabolic engineering studies.

**Results:**

In this study, P_trpC_, a kind of promoter with a relatively appropriate expression level and small size, was selected as a monomer for promoter library construction. Through in vitro BioBricks assembly, 9 overlapping P_trpC_ promoters with different copy numbers as well as reporter gene *gfp* were connected and subsequently integrated into the genome of *C. militaris*. Both the mRNA transcription level and the expression level of gene *gfp* gradually increased along with the copy number of the overlapping promoter NP_trpC_ and peaked at 7. In the meantime, no significant difference was found in either the biomass or morphological characteristic of engineered and wild-type strains*.*

**Conclusions:**

This study firstly expanded the overlapping promoter strategy used in model microorganism in *C. militari*s. It was a proof-of-concept in fungi synthetic biology and provide a general method to pushed the boundary of promoter engineering in edible mushroom.

**Supplementary Information:**

The online version contains supplementary material available at 10.1186/s12934-022-01826-0.

## Introduction

*Cordyceps militaris* is generally regarded as an edible and medicinal fungus in East Asia. It has recently been widely reported due to its diverse bio-active component (e.g., cordycepin [1], polysaccharide [2], carotenoid [3]), which have been proved to have anti-cancer, anti-tumor [4, 5], and anti-photoaging [6, 7] effects. It is the only species that can produce a large amount of cordycepin among more than 350 species of the *Cordyceps* genus. Artificial cultivation techniques of *C. militaris* have been developed for more than 40 years and most parameters (e.g., culture medium composition, culture conditions) have been fully optimized [8–10]. By 2015, the output of large-scale industrialized planting of *C. militaris* in China has reached 74,000 tons [11].

Diverse synthetic biology systems have been recently developed in *C. militaris*. In 2018, gene editing techniques based on homologous recombination [12] and CRISPR [13] were established in *C. militaris*, which made it possible to reconstruct its cordycepin metabolic network. What’s more, the genome metabolism model iNR1329 allowed genome-wide in silico analysis of gene of interest (GOI) in *C. militaris* [14]. For another, the study of setting up *C. militaris* as microbial cell factory boomed in recent years with the rapid development in synthetic biology. Zou et al.used *C. militaris* as chassis cells to transform them into a cell factory with high-yield anticancer drug pentostatin [15]. Since the industrial cultivation techniques for various mushroom was mature, there is growing interest in such attempts to reconstruct mushroom species to overproduce their valuable bio-active compound, and on a more and more complexity level.

Promoter engineering was the most popular and convenient strategy to regulate the expression level of GOI [16, 17]. And constitutive promoter present stable expression level, which was essential to accurate regulation. However, the poor DNA element diversity has limited the present development of fungi synthetic biology to achieve a fixed ratio of multiple gene expression levels. So far, there are only four kinds of constitutive promoters (e.g., P_gpd_ [18], P_trpC_ [19], P_cmlsm3_ [13], P_CaMV_ [20]) reported in *C. militaris*. With limited available promoter on hand, the overlapping constitutive promoter was a practicable strategy widely applied in edible mushroom. And BioBricks method was capable to construct gene cluster repeatedly and sequentially by reusing isocaudarners with similar restriction sites [21]. Constitutive promoter P_trpC_ [19] regulated the glyceraldehyde 3-phosphate dehydrogenase gene in *C. militaris* was chosen to be overlapped due to its small size and medium expression strength (Additional file 1: Table S1). 1 to 9 P_trpC_ were overlapped by BioBricks method, ligated to reporter gene *gfp* and each module was integrated into the genome of *C. militaris* strain CM10 by *Agrobacterium*-mediated transformation (ATMT) respectively. Both mRNA and fluorescence intensity increased with the copy number of promoters at the beginning and presented a normal distribution as a whole. This study proved the overlapping promoter strategy worked well in fungi and provided a new path to achieve rational balance among multiple GOI in mushrooms.

## Results

### Construction of pMD19T (simple)-1-9P_trpC_-***gfp***-T_nos_

The size of promoter P_trpC_ was only 369 bp, which was much smaller than other constitutive promoters (Additional file 1: Table S1) discovered in *C. militaris*. Therefore, it was chosen to construct an overlapping promoter library with 1–9 copies to achieve quantitative expression of multiple target genes.

**Fig. 1:**
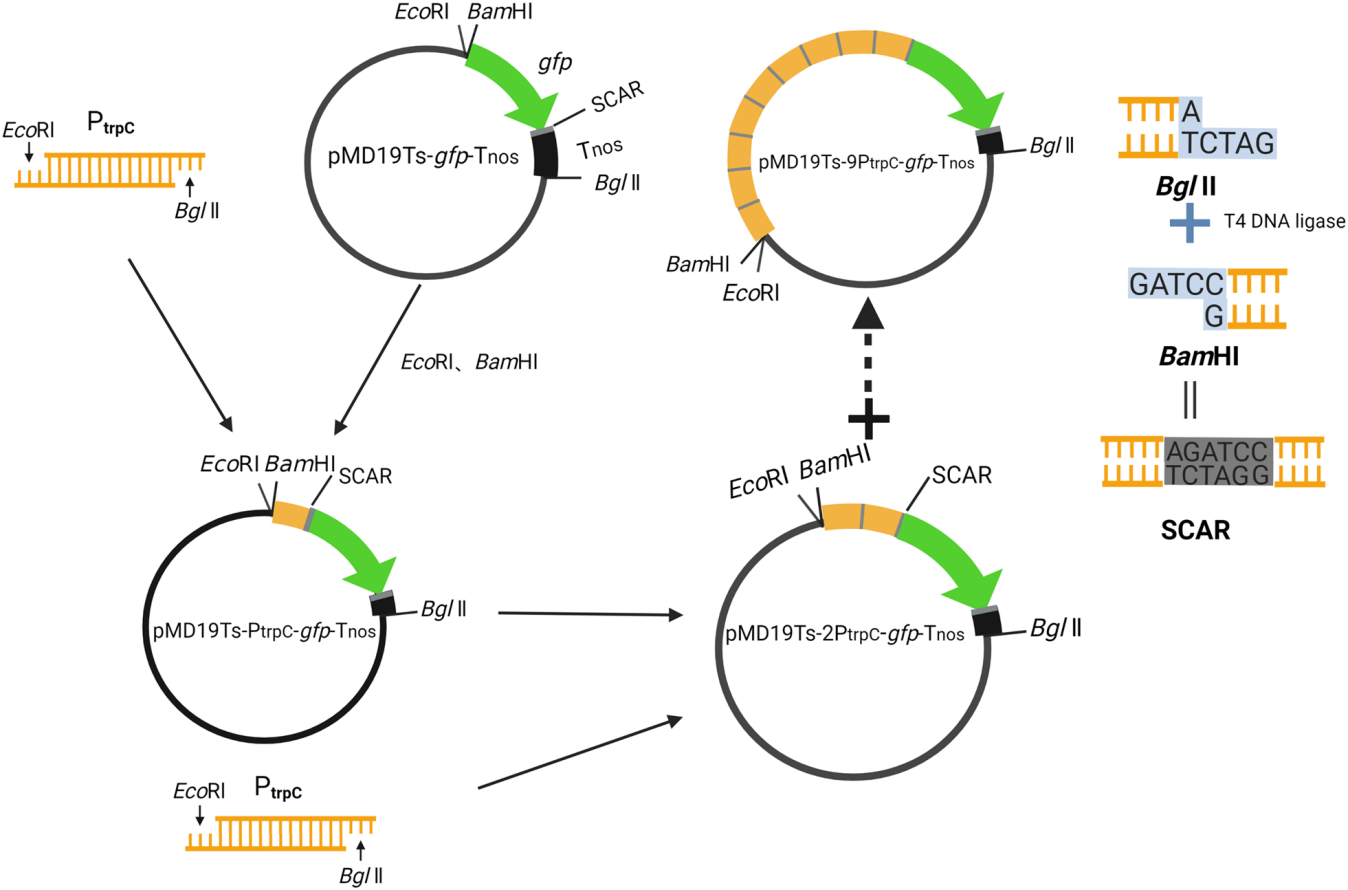
Flow diagram of pMD19T(simple)-1-9P_trpC_-*gfp*-T_nos_ vector construction

**Fig. 2:**
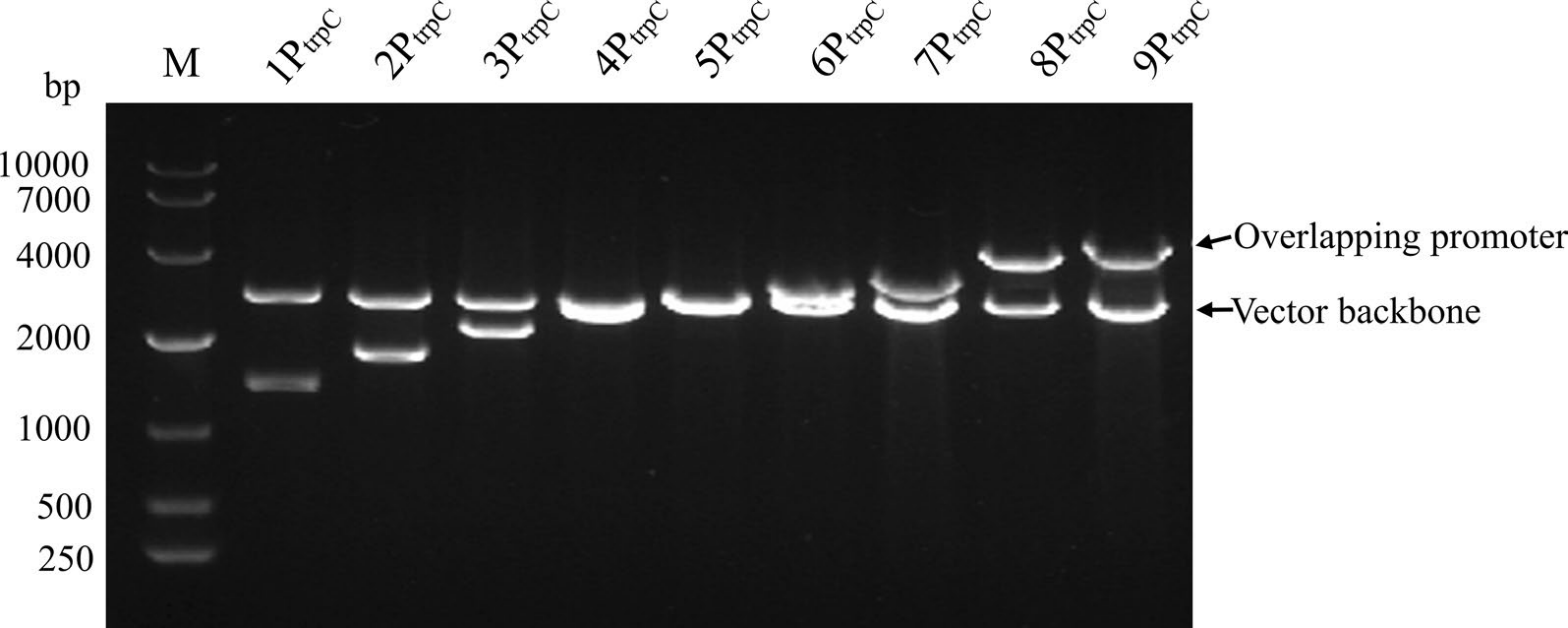
Restriction enzyme identification of pMD19T(simple)-1-9P_trpC_-*gfp*-T_nos._ (M: DNA marker, 1–9 were: pMD19T(simple)-1-9P_trpC_-*gfp*-T_nos_, respectively)

After 4 rounds of BioBricks assembly, vectors pMD19T(simple)-1-9P_trpC_-*gfp*-T_nos_ were constructed (Fig. 1). When these 9 promoters were digested by *Eco*RI and *Bgl*II, the remaining vector backbones should be 2705 bp and the operon with overlapping promoters should be 1362, 1737, 2112, 2487, 2862, 3237, 3612, 3987, and 4362 bp respectively. The experimental result was consistent with the theoretical result (Fig. 2).

### Construction of pCambia0390-*blpR*-1-9P_trpC_-*gfp*

**Fig. 3:**
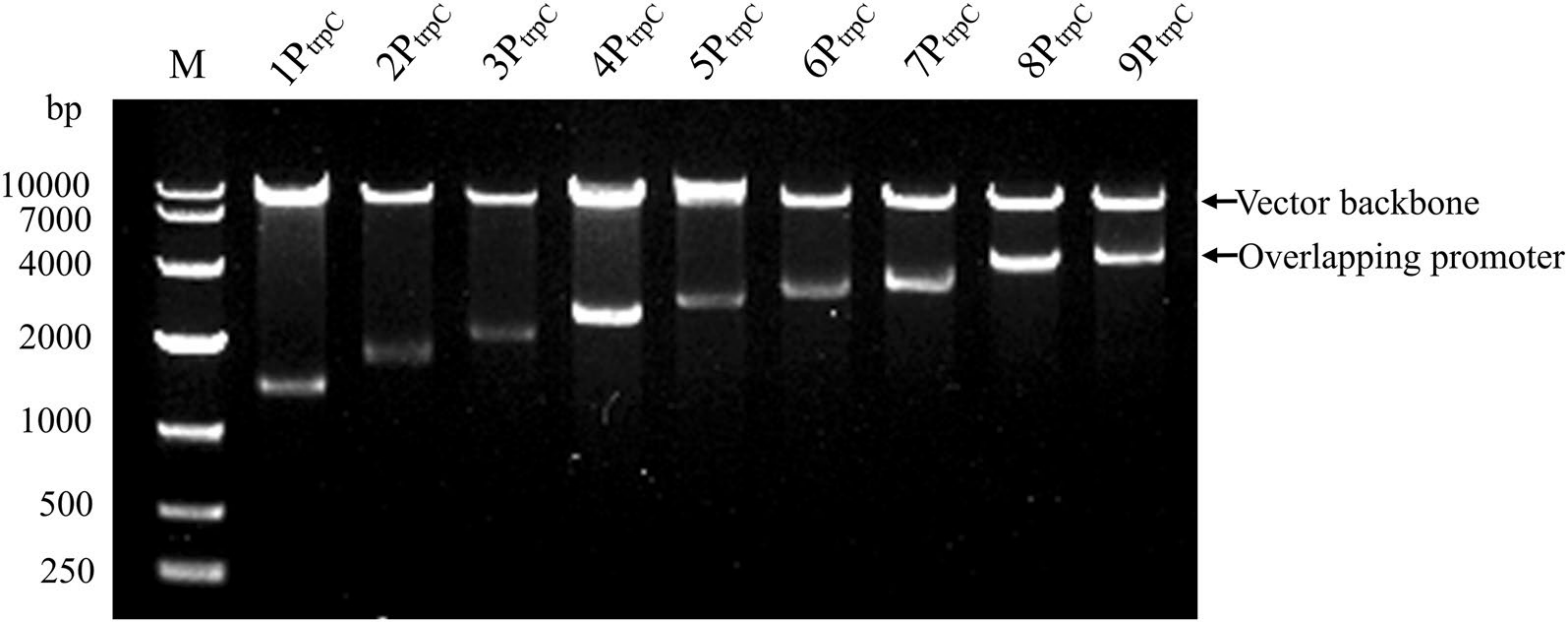
Restriction enzyme digestion identification of pCambia0390-*blpR*-1-9P_trpC_-*gfp*. Vector pCambia0390-*blpR*-1-9P_trpC_-*gfp* were double digested (M: DNA marker; 1–9: pCambia0390-*blpR*-1-9P_trpC_-*gfp*, respectively)

The cassettes 1-9P_trpC_-*gfp*-T_nos_ cut from vectors pMD19T(simple)-1-9P_trpC_-*gfp*-T_nos_ were inserted into shuttle vector pCambia0390 respectively. They were separated by *Eco*RI and *Bgl*II into an 8109 bp vector backbone and a 1362, 1737, 2112, 2487, 2862, 3237, 3612, 3987, and 4362 bp operon respectively. The experimental result was consistent with the theoretical result (Fig. 3).

**Fig. 4:**
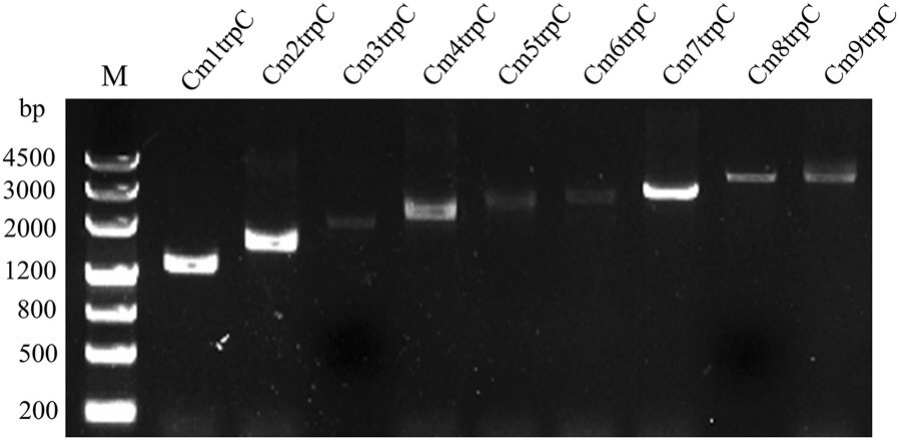
PCR identification of recombinant transformants of *C. militaris*. (M: DNA marker; 1–9: Cm1-9trpC, respectively)

**Fig. 5:**
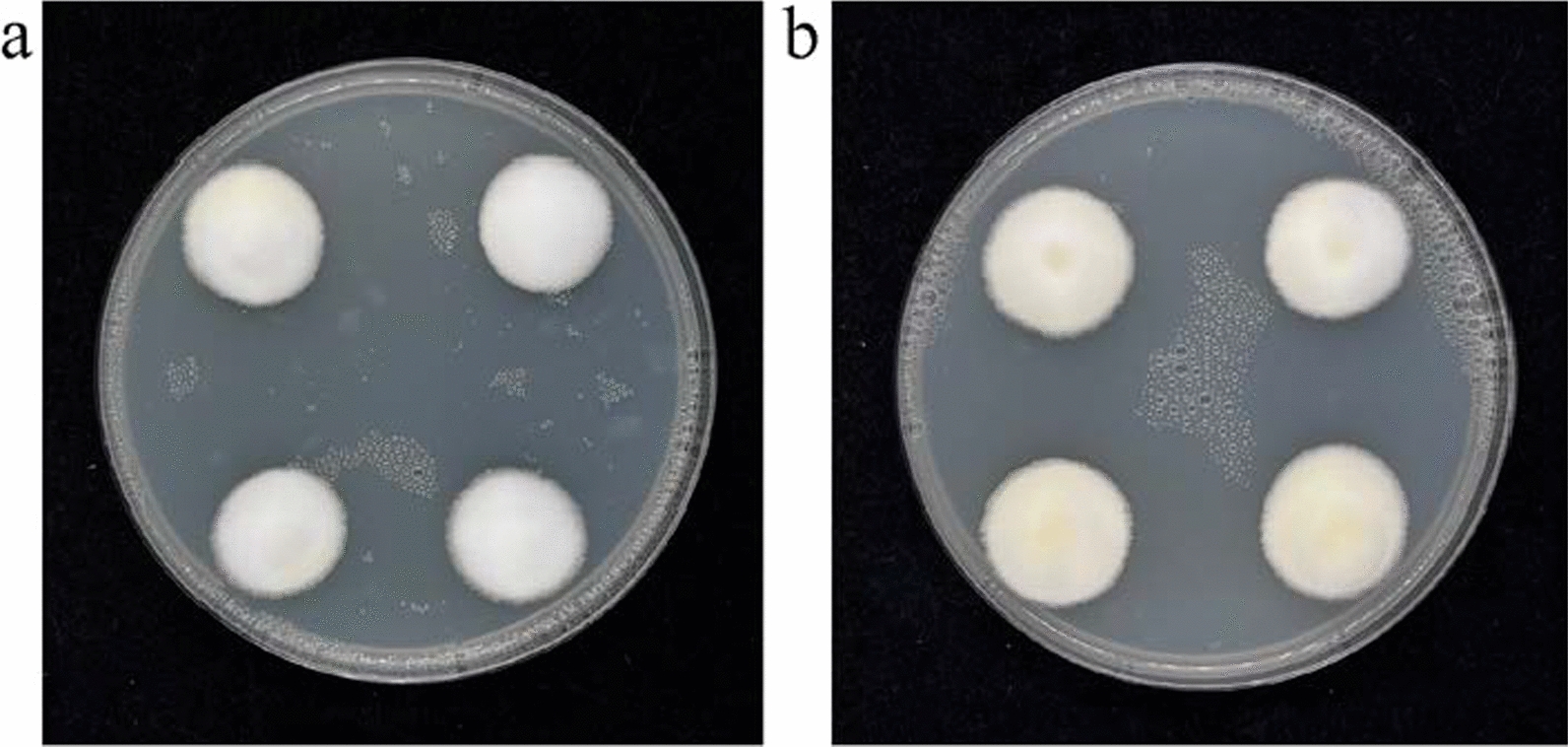
Mycelia of *C. militaris* grown on plates. a, the wild-type strains; b, the recombinant strains

Colony PCR results showed that vectors pCambia0390-*blpR*-1-9P_trpC_-*gfp* were successfully transferred into *A. tumefaciens* strain AGL-1 by electro-transformation. And recombinant *C. militaris* hyphae infected by *A. tumefaciens* AGL_ pCambia0390-blpR-1-9P_trpC_-*gfp* were scraped and identified by PCR (Fig. 4). Since the robustness of fungi recombinant was relatively low, the obtained resistant transformed strains were continuously sub-cultured and further identified by PCR (Fig. 4). The stable strains were those with purposeful bands at the sizes of 1325, 1700, 2075, 2450, 2825, 3200, 3575, 3950, and 4325 bp respectively. The 3rd generation of stable recombinants was named as CmNtrpC and selected for subsequent experiments. Result showed that no significant difference in mycelia was found between wild-type and recombinant strains (Fig. 5a and b).

### Stable expression of gene *gfp* in* C. militaris*

**Fig. 6:**
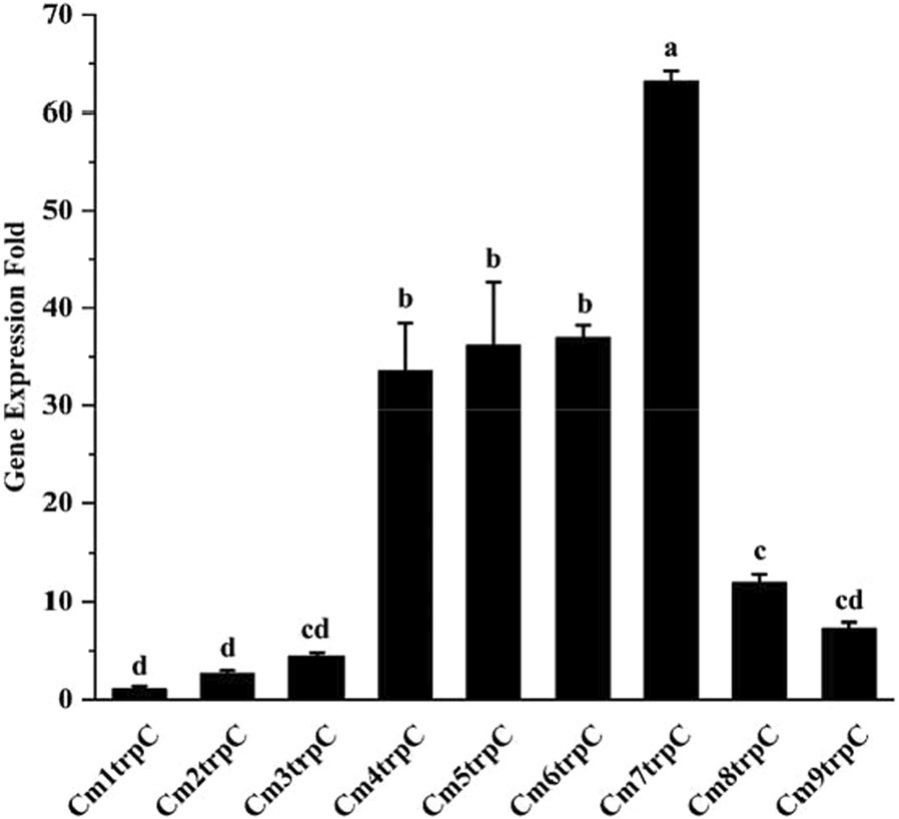
qRT-PCR detection of gene *gfp* in transformant strains. The gene expression of Cm1trpC in control is normalized to 1. The data is the average of three experiments, and the error line shows the standard error. T-test was used for statistical analysis (*p* < 0.05).

The transcription levels of gene *gfp* in CmNtrpC were detected by qPCR and the mycelial fluorescence intensities were investigated by laser confocal microscope. The ratio of transcription level of engineered strains to Cm1trpC increased with the copy number of overlapping promoters P_trpC_ and peaked at 7 (Fig. 6). Compared with strain Cm1trpC, the transcription level of strain Cm7trpC increased 63 folds. The transcription levels of strains Cm4-7trpC were relatively high while the others were significantly lower. This result indicated that the transcription strength can be enhanced by increasing the tandem repeats of the P_trpC_ promoter. However, the increase was in a normal distribution. And even the copy number increased from 1 to 7, the increase of fluorescence intensity was not linear.

**Fig. 7:**
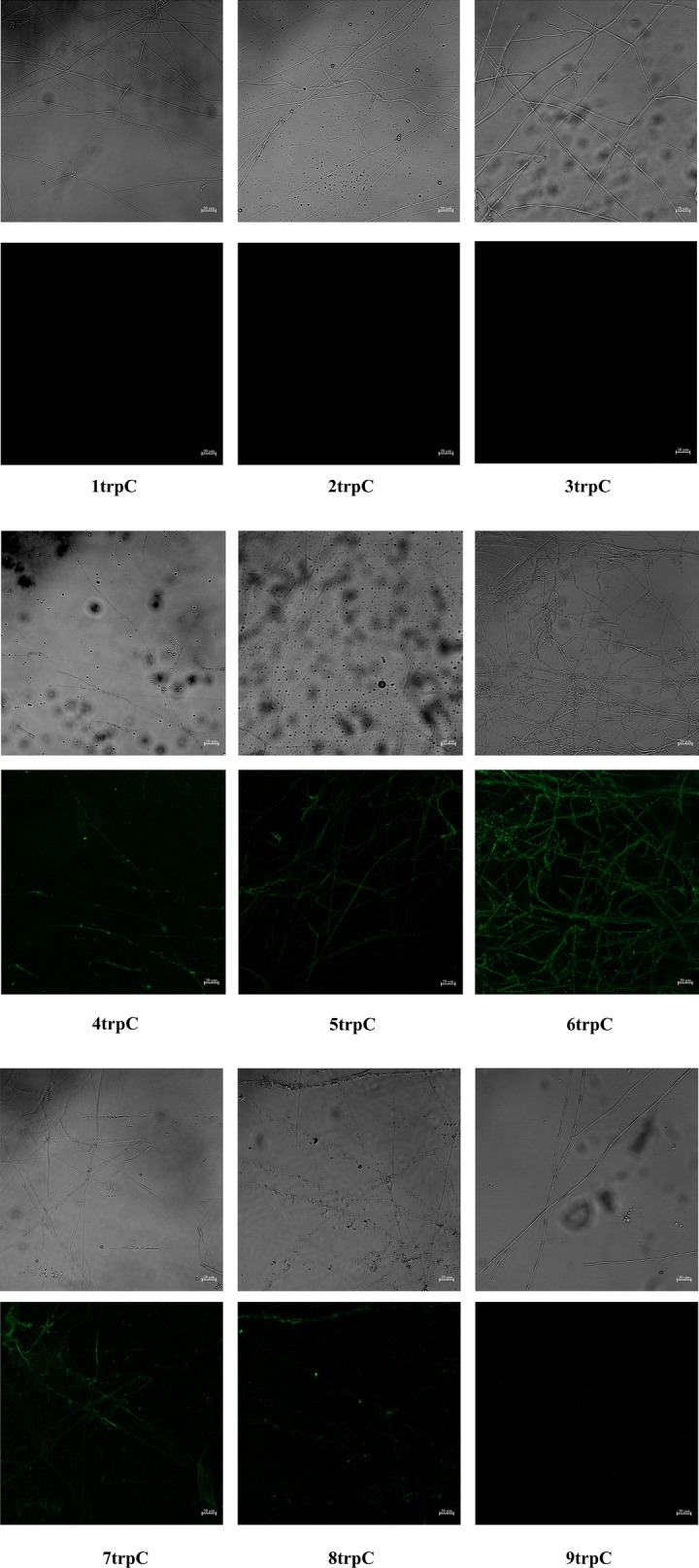
Fluorescence intensities of GFP in transformant mycelia. Mycelia of the Cm1-9trpC transformants were shown under bright field image and fluorescence microscope (excitation, 395 to 440 nm, and emission, 470 nm). Scale bars represent 20 µm

The protein expression level was basically consistent with the result of transcription level. The fluorescence intensity of a single mycelium gradually increased with the copy number of overlapping promoters (Fig. 7 and Additional file 1: Table S3). It also peaked when the copy number reached 7. The fluorescence intensities of strains Cm4-7trpC were relatively higher while the others were too low to be captured by software ImageJ. The fluorescence intensity values of remaining strain Cm4-8trpC showed similar fold change. For example, the mRNA transcription level of Cm7trpC was 1.7-fold higher than Cm6trpC, while fluorescence intensity was 1.6-fold. Results of both mRNA level and fluorescence intensity indicated that the expression level of the target gene could be enhanced with the increase of overlapping promoter number. The increase was not limitless, and it would usually reach a boundary with the increase of promoter copy number.

**Fig. 8:**
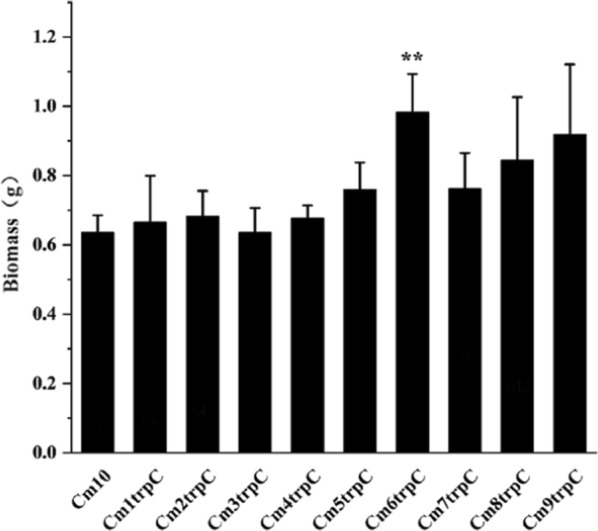
Biomass of wild-type and recombinant strains of *C. militaris*. The data is the average of three experiments, and the error line shows the standard error. *It indicates that the biomass of recombinant strain is significantly higher when it was compared with that of wild-type strain, *p < 0.05, ** *p* < 0.01

The biomass of both wild-type strain and engineered strains CmNtrpC were measured. Compared with the wild-type strain CM10, the biomass of engineered strains slightly increased with the copy number of promoters (Fig. 8), indicating that the integration of gene *gfp* module with multiple overlapping promoters would not burden the growth of *C. militaris*. Furthermore, biomass data, growth rate, and biomass productivity, which were key indexes of cell growth, showed no significant difference (Additional file 1: Table S2). And since strain CM10 was degenerated and lost its capability to form fruiting body, it was necessary to compare the morphological characteristic of fruiting body when similar promoter library was integrated in an undegenerated strain.

## Discussion

In this study, modules with 9 overlapping promoters were constructed by the BioBricks method and integrated into the edible fungus *C. militaris* via ATMT. The result of transcription level detected by RT-PCR was well correlated with the result of fluorescence intensity detected by confocal microscope. The initiation intensity of reporter gene *gfp* showed a normal distribution while the initiation intensity reached the highest level with 7 monomer promoters. The pattern was consistent with the previous report in *E. coli* [16]. It decreased when the copy number of promoters passed a particular singularity. It might be may be due to the transcription interference caused by the connection between RNA polymerases and tandem promoters [22]. Yet, it takes further experimental methods, e.g., ChIP, to dig out the truth.

Furthermore, even though this study explored the pattern of overlapping P_trpC_ promoter, the standard of monomer promoter (e.g., minimum and maximum size, strength, DNA sequence characteristic) was still unclear. Previous study reported that promoter P_37_ with the size of only 55 bp was a practicable candidate for overlapping promoter engineering in *E. coli* [23]. Therefore, the minimum size of the repetitive promoter worked in *C. militaris* might be much smaller than the size of promoter P_trpC_. Kanamasa et al. concatenated the promoter region III with 12 copies and used it to enhance the expression of β-Mannosidase in *Aspergillus echinosporus* [24]. What’s more, Zhang et al. overlapped 4 copies of cbhI inducible promoter to improve the expression level of the β-glucosidase gene (*bgl1*) in *Trichoderma reesei*) [25]. The size of promoter cbh1 was larger than 1291 bp. It implied that the maximum size of the monomer promoter could be much larger than the promoter we used in this study. And not only constitutive promoter but inducible promoter could be applied as a candidate in overlapping promoter strategy. For another, the carrying capacity of the general plasmid was limited and usually no more than 50 kb. The maximum size of the monomer promoter might be limited by the current molecular cloning method. Considering the convenience of molecular cloning and better binding between RNA polymerase and promoter sequence, a hypothesis for overlapping promoter strategy in filamentous fungi was provided in this study: a small promoter with medium strength would be a better candidate. Therefore, frequently used promoters in filamentous fungi (e.g., P_oliC_ with only 212 bp, and P_acuD_ with only 286 bp [26]) might be a practical choice.

Diverse metabolic engineering strategies in *C. militaris*, including CRISPR and split marker homologous recombination to edit genomic DNA as well as genome model iNR1329 to perform in silico metabolic analysis, were developed in recent years [12–14]. To make full use of these strategies and further develop fungus synthetic biology, it was essential and urgent to expand the promoter database and achieve accurate gene expression. And to rebalance the metabolic flux of different pathways, it was common to use promoters with different intensities to regulate and control the expression of multiple genes [27]. However, it was knotty to develop diverse promoters with different intensities in undeveloped mushroom species. Previous research generally mutated homologous promoters [28] or utilized heterogeneous promoters, which were either laborious or violate the principle of gene editing products [29]. This research aided the rational construction of promoter library with different intensities in undeveloped mushroom species and provided a one-step method for heterogeneous expression with diverse strength in *C. militaris* without overusing promoter sources, which saves more available choices for additional editing. It also proved that it was feasible to transform the synthetic biology strategy developed in model species to non-model fungus.

The engineered strains CmNtrpC were sub-cultured and tested by PCR for 3 generations. No significant degeneration was observed. With the addition of applying natural selection and culture preservation, the natural problem of *C. militaris* degeneration was soluble. Furthermore, even though the molecular mechanism of *C. militaris* degeneration remained unclear [30], the overlapping promoter strategy has provided one-step repetitive construction of engineered *C. militaris* strain with fix expression ratio of multiple target strains. Once the engineered strains were degenerated, they could be quickly reconstructed by re-invasion of engineered *Agrobacterium* strain AGL-1_pCAMBIA0390-NPtrpC-GOI to any wild-type *C. militaris* strain. The *Agrobacterium* strain rarely degenerated and the constructed vector was highly stable.

Compared with model species with clear genetic background and diverse promoter elements, such as *E. coli* and *Saccharomyces cerevisiae*, *C. militaris* was the Robinson Crusoe island with restricted choices. Even though promoter P_trpC_ had appropriate moderate intensity and relatively small size, it was still way too larger than the promoter chosen in the overlapping promoter research in *E. coli* [16, 23]. The combination of 9 P_trpC_ promoters was about 3.2 kb and larger than general gene. It made the library lack flexibility in vector construction and module integration. With more and more in silico tools developed for promoter prediction, it was still necessary to discover promoters with smaller sizes and appropriate starting strength in the future.

## Materials and methods

### Strains and cultivation conditions

*E. coli* strain DH5α (stored in our laboratory) was used in the construction and transformation of vectors. *A. tumefaciens* strain AGL-1(Weidi Bio, Shanghai, China) and the pCAMBIA0390 vector (Cambia, Queensland, Australia) were used for fungal transformation. *C. militaris* strain CM10 was purchased from Haixin Biological Co., LTD (Shandong, China) as the host for gene disruption. Luria- Bertani medium was used for the cultivation of *E. coli* strain DH5α and *A. tumefaciens* strain AGL-1. *C. militaris* strain CM10 was cultured in potato peptone dextrose agar (PDA: 200 g/L potatoes, 20 g/L glucose, 3 g/L KH_2_PO_4_, 1.5 g/L MgSO_4_, 20 g/L agar)) at 25℃. The materials for the strains and plasmids were shown in Table 1.

**Table 1:** Materials of strains and plasmids

Strains/plasmids	Source
*Escherichia coli* strain DH5α	This lab
*Agrobacterium tumefaciens* AGL-1	Purchase from Weidi Bio, Shanghai, China
*Cordyceps militaris* strain CM10	Purchase from Haixin Bio, Shandong, China
pP_rstA_-GFP	Given by Prof. Jianzhong Liu from Sun Yat-Sen University
pAg1-H3	Given by Prof. Gang Liu and Prof. Yuanyuan Pan from Institute of Microbiology, Chinese Academy of Sciences
pCambia0390-*blpR*	This lab
pMD19T(simple)-1-9P_trpC_-*gfp*-T_nos_	This work
pCambia0390-*blpR*-1-9P_trpC_-*gfp*-T_nos_	This work

### Plasmid construction

#### Construction of pMD19T (simple)-1-9P_trpC_-*gfp*-T_nos_

The terminator T_nos_ was cloned from pCambia0390 by forward primer Tnos-F with *Eco*RI and *Bam*HI restriction sites and reverse primer Tnos-R with *Bgl*II restriction site to build up BioBricks adaptor. And it was linked to vector pMD19T(Simple)(Takara, Beijing, China) by TA cloning and transformed into the competent cell of *E. coli* strain DH5α prepared by 0.1 M CaCl_2_. The recombinant was identified by colony PCR and DNA sequencing from transformants.

Gene *gfp* coding green fluorescent protein was amplified by PrimeSTAR Max (Takara, Beijing, China) with vector pP_rstA_-GFP as template and GFP-F/R as primer. *Eco*RI-*Bam*HI cleavage site and protective base were added to forward primer GFP-F, and *Bgl*II cleavage site and protective base were added to reverse primer GFP-R. Gene *gfp* fragment and vector pMD19T(simple)-T_nos_ were digested by isocaudarner *Bam*HI and *Bgl*II and further linked by T4 DNA ligase (Takara, Beijing, China) to generate a scar sequence (AGATCC). The linked product was transformed into strain DH5α and the transformant was identified.

Promoter P_trpC_ was amplified largely by the same method that performed to gene *gfp*. Vector pAg1-H3 was used as template and trpC-F/R was used as primer (Table. 2). What’s more, more copies of promoter P_trpC_ were inserted and vector library containing pMD19T(simple)-2–9-P_trpC_-*gfp*-T_nos_ were constructed by the BioBricks method.

**Table 2:** Primers for PCR and qPCR

Usages	Primers	Sequences (5'-3')
PCR verification	Tnos-F	CCGAATTCTTGGATCCGATCGTTCAAACATTTGGCA
	Tnos-R	CCCAGATCTGATCTAGTAACATAGATGAC
	GFP-F	CCGAATTCTTGGATCCAGTAAAGGAGAAGAACTTTT
	GFP-R	CCCAGATCTCTATTTGTATAGTTCATCC
	trpC-F	CCGAATTCTTGGATCCTCGACAGAAGATGATATTGAA
	trpC-R	CCCAGATCTATCGATGCTTGGGTAGAATA
	M13F	GTAAAACGACGGCCAGT
	M13R	CAGGAAACAGCTATGAC
	Tu3-F	CATTCGCATCGTCATTGTTGGCTC
qPCR analysis	18S rRNA-F	GAGCCCAAGCACTTTGATTTCT
	18S rRNA-R	GCATTTGCCAAGGATGTTTTC
	qPCR-GFP-F	AGTTGTCCCAATTCTTGTTG
	qPCR-GFP-R	TGTCTTGTAGTTCCCGTCA

The underline indicated restriction site

#### Construction of pCambia0390-*blpR*-1-9P_trpC_-*gfp*-T_nos_

To achieve *Agrobacterium-*mediated transformation, shuttle vector pCambia0390-*blpR* was chosen for the integration of the repetitive P_trpC_ promoter library. Digested by *Eco*RI and *Bgl*II, an 8109-bp linear vector backbone was purified by a DNA Gel Extraction Kit (Magen, Beijing, China). To further separate the operon with overlapping promoters, vectors pMD19T(simple)-1-3P_trpC_-*gfp*-T_nos_ and pMD19T(simple)-7-9P_trpC_-*gfp*-T_nos_ were digested by *Eco*RI and *Bgl*II, thereinto, *Sca*I was additionally added to digest backbone vector and obtain NP_trpC_-*gfp-*T_nos_ module since the size of restriction fragments of pMD19T(simple)-4-6P_trpC_-*gfp*-T_nos_ were similar, while pMD19T(simple)-4-6P_trpC_-*gfp*-T_nos_ were digested by *Eco*RI, *Bgl*II, and *Sca*I. The operon with overlapping promoters with sizes of 1362, 1737, 2112, 2487, 2862, 3237, 3612, 3987, and 4362 bp was respectively purified. Ligation with overlapping promoters was transformed. Vectors pCambia0390-*blpR*-1-9P_trpC_-*gfp*-T_nos_ were obtained by colony PCR and enzyme digestion.

### *Agrobacterium*-mediated transformation

*A. tumefaciens*-mediated transformation(ATMT) was used as described in a previous study [13]. Shuttle vectors pCambia0390-*blpR*-1-9P_trpC_-*gfp*-T_nos_ were transformed to the competent cells of *A. tumefaciens* strain AGL-1. Recombinant was re-inoculated in 5 mL LB medium (with kanamycin 50 mg/L and carbenicillin 50 mg/L) at 30 ℃ for 2–3 days to obtain seed solution. It was further inoculated into IM medium (1.45 g/L KH_2_PO_4_,2.05 g/L K_2_HPO_4_, 0.15 g/L NaCl, 0.5 g/L MgSO_4_·7H_2_O, 66 mg/L CaCl_2_·2H_2_O, 2.48 mg/L FeSO_4_·7H_2_O, 0.5 g/L (NH_4_)_2_SO_4_, 1.8 g/L Glucose, 5 mL/L Glycerin, pH 5.5, with 200 μM AS) with initial 0.15 OD_600_ value and kept in suspension culture at 30℃ till the OD_600_ value reached 0.8. *A. tumefaciens* suspension preheated at 25℃ and *C. militaris* conidia suspensions (1 × 10^4^ conidia mL^−1^) were mixed in diverse certain proportions (1:100, 1:1, 100:1). Mixture was separated on IMA solid medium (1.45 g/L KH_2_PO_4_,2.05 g/L K_2_HPO_4_, 0.15 g/L NaCl, 0.5 g/L MgSO_4_·7H_2_O, 66 mg/L CaCl_2_·2H_2_O, 2.48 mg/L FeSO_4_·7H_2_O, 0.5 g/L (NH_4_)_2_SO_4_, 0.9 g/L Glucose, 5 mL/L Glycerin, 15 g/L Agar, pH 5.5, with 200 μM AS, pH = 5.5) pasted with glass paper (diameter = 90 mm) and cultured in the dark at 25℃ for 3 days. Glass paper was transferred to PDA medium with cephalosporin (300 mg/L) and Basta (400 mg/L) for 7 days when spores were germinated and white spots were emitted. Once was visible, a single mycelial mass was picked it by toothpick and transferred to the potato PDA with Basta (400 mg/L). When a single mycelium grows to 2 cm in diameter, cut 0.5 cm in diameter for PCR verification of crude mycelium samples. Verified transformants were re-cultivated till passed to the third generation. Mycelia were collected to extract genomic DNA and perform PCR analysis with primer pairs Tu3-F/GFP-R. Putative transformants were tested by quantitative real-time PCR and confocal laser scanning microscope.

### qPCR verification of gene *gfp* transcription level initiated by overlapping promoters

Transformants with overlapping promoters were inoculated in liquid PDB media at 25 ℃ and 150 rpm for about 7 days. Mycelium was washed with ddH_2_O 3 times and ground with liquid nitrogen. 100 mg powder was separated to extract total RNA with E.Z.N.A. Fungal RNA Miniprep kit (OMEGA Bio-Tek Inc., GA, USA). 1 µg RNA was taken and reverse transcribed into cDNA by HiScript III-RT SuperMix (Vazyme, Nanjing, China). 50 ng of cDNA, 0.2 μL each of relevant primers (Table. 2) and SYBR qPCR Master Mix (Vazyme, Nanjing, China) were combined to build up the 20-µL qPCR system. ABI 7500 Real-Time PCR System (Thermo Fisher Scientific, MA, USA) and 18S rRNA gene as the internal control gene [31] were used for Quantitative Real-Time PCR. The cDNA of wild-type strain CM10 and engineered strains Cm1-9trpC were used as templates and 18S rRNA-F/R and GFP-F/R were used as primers for qRT-PCR amplification. Taking wild-type strain CM10 as the negative control and the *gfp* gene expression of *C. militaris* Cm1trpC as the control, the relative transcription level of gene *gfp* in other 8 strains were calculated respectively. The relative transcription of mRNA was computed by 2^−ΔΔCT^ (livak) [32].

### Observation of fluorescence *C. militaris* by confocal laser scanning microscope

Dry and sterilized cover glass (20 mm × 20 mm) was inserted into the PDA medium inoculated with corresponding transformant blocks at an angle of 45°. Transformant was subsequently cultured at 25℃ till mycelium grew beyond the insertion position. And the cover glass was taken out for observation in Carl Zeiss LSM800 light and fluorescence microscope (Carl Zeiss AG, Oberkochen, Germany). The mycelial fluorescence of engineered strains was observed and the fluorescence intensity of wild-type strain CM10 was regarded as the negative control to remove the influence of its own fluorescence background.

## Supplementary Information

**Additional file 1: Table S1.** Basic parameters of the promoter commonly used in *Cordyceps militaris*. **Table S2.** Biomass, growth rate, and biomass productivity of wild-type and recombinant strains. **Table S3.** Fluorescence intensities of GFP in transformant mycelia. **Figure S1.** Plasmid map of pCambia0390-*blpR*-9P_trpC_-*gfp*.

## Data Availability

All data generated or analyzed during this study are included in this published article and its additional files.
