# Supplementary material for: Overlapping promoter library designed for rational heterogenous expression in Cordyceps militaris
Source: Microb Cell Fact. 2022 Jun 2;21:107. doi: 10.1186/s12934-022-01826-0 (PMC9161592; doi:10.1186/s12934-022-01826-0)
Supplement: Supplementary file 1 — Additional file 1: Table S1. Basic parameters of the promoter commonly used in Cordyceps militaris. Table S2. Biomass, growth rate, and biomass productivity of wild-type and recombinant strains. Table S3. Fluorescence intensities of GFP in transformant mycelia. Figure S1. Plasmid map of pCambia0390-blpR-9PtrpC-gfp. [file 12934_2022_1826_MOESM1_ESM.docx]

Additional file 1: Table S1 Basic parameters of the promoter commonly used in *Cordyceps militaris.*

| Name | Size(bp) | Type | Describe |
| --- | --- | --- | --- |
| P_trpC_ | 369 | constitutive promoter | Promoter of gene *trpC* from *Aspergillus nidulans*, medium strength |
| P_cmlsm3_ | 547 | constitutive promoter | Promoter of U6 small nuclear ribonucleoprotein gene, medium strength |
| P_CaMV_ | 678 | constitutive promoter | Promoter from 35S RNA, medium strength |
| P_gpd_ | 1035 | constitutive promoter | Promoter of glyceraldehyde 3-phosphate dehydrogenase gene |

Additional file 1: Table S2 Biomass, growth rate, and biomass productivity of wild-type and recombinant strains

| Name | Biomass(mg) | Growth Rate(mg/d) | Biomass Productivity(mg/mL) |
| --- | --- | --- | --- |
| Cm10 | 635±51 | 79±6 | 6.35±0.51 |
| Cm1trpC | 665±135 | 83±2 | 6.65±1.35 |
| Cm2trpC | 682±73 | 85±9 | 6.82±0.73 |
| Cm3trpC | 635±72 | 79±9 | 6.35±0.72 |
| Cm4trpC | 677±37 | 84±5 | 6.77±0.37 |
| Cm5trpC | 760±78 | 95±10 | 7.60±0.78 |
| Cm6trpC | 983±111 | 123±14 | 9.83±1.11 |
| Cm7trpC | 762±103 | 95±13 | 7.62±1.03 |
| Cm8trpC | 844±183 | 105±23 | 8.44±1.83 |
| Cm9trpC | 918±203 | 115±25 | 9.18±2.03 |

Additional file 1: Table S3 Fluorescence intensities of GFP in transformant mycelia

| Name | Fluorescence intensity (AU) |
| --- | --- |
| Cm4trpC | 13.99±0.13 |
| Cm5trpC | 14.48±1.27 |
| Cm6trpC | 17.96±0.86 |
| Cm7trpC | 28.18±4.92 |
| Cm8trpC | 5.31±0.21 |

Note: Cm1-4trpC is not included in the table because the fluorescence value is too low.


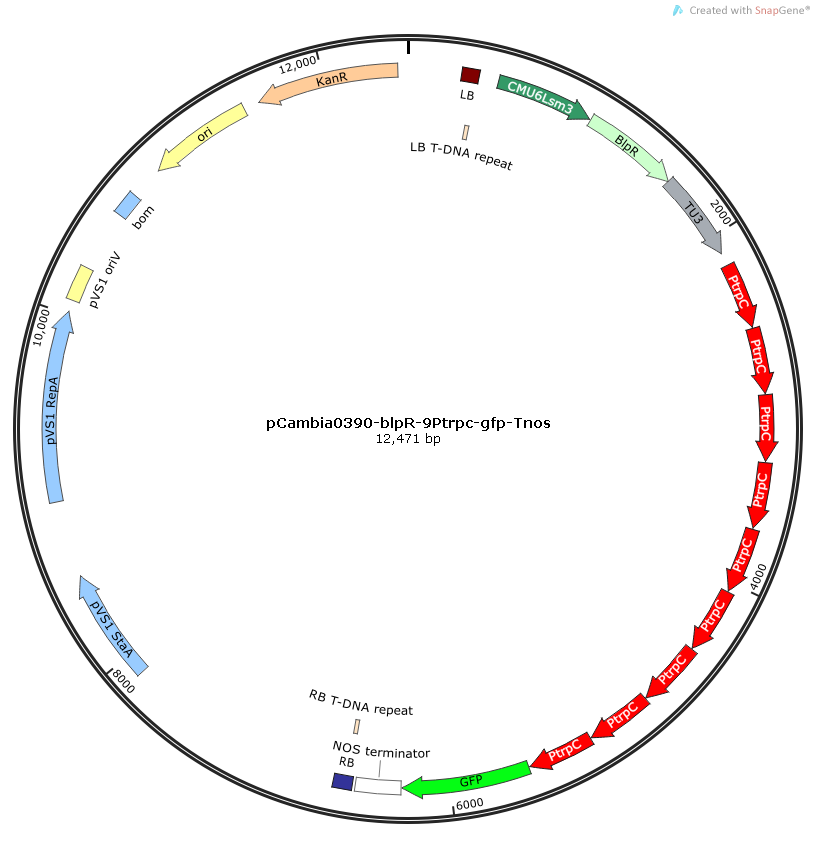


Additional file 1: Fig S1 Plasmid map of pCambia0390-*blpR*-9P_trpC_-*gfp*.
